# Prospects for Irradiation in Cellulosic Ethanol Production

**DOI:** 10.1155/2015/157139

**Published:** 2015-12-29

**Authors:** Anita Saini, Neeraj K. Aggarwal, Anuja Sharma, Anita Yadav

**Affiliations:** ^1^Department of Microbiology, Kurukshetra University, Kurukshetra, Haryana 136119, India; ^2^Department of Biotechnology, Kurukshetra University, Kurukshetra, Haryana 136119, India

## Abstract

Second generation bioethanol production technology relies on lignocellulosic biomass composed of hemicelluloses, celluloses, and lignin components. Cellulose and hemicellulose are sources of fermentable sugars. But the structural characteristics of lignocelluloses pose hindrance to the conversion of these sugar polysaccharides into ethanol. The process of ethanol production, therefore, involves an expensive and energy intensive step of pretreatment, which reduces the recalcitrance of lignocellulose and makes feedstock more susceptible to saccharification. Various physical, chemical, biological, or combined methods are employed to pretreat lignocelluloses. Irradiation is one of the common and promising physical methods of pretreatment, which involves ultrasonic waves, microwaves, *γ*-rays, and electron beam. Irradiation is also known to enhance the effect of saccharification. This review explains the role of different radiations in the production of cellulosic ethanol.

## 1. Introduction

**Figure 1:**
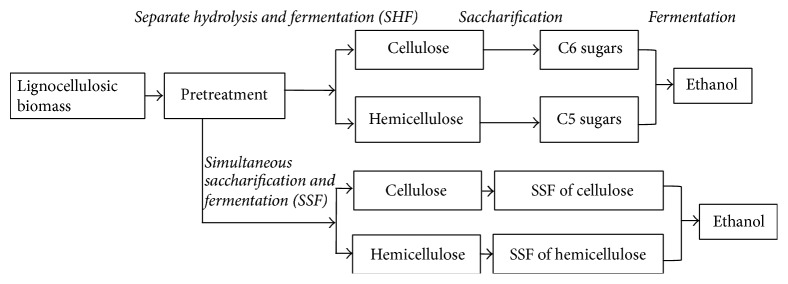
Scheme of production of cellulosic ethanol.

Rapid exploitation of the energy sources has led to their depletion [1] causing the problem of energy security to the future generations. Limited availability of the fossil fuels and long duration involved in their production have necessitated the search for renewable sources of energy. Biofuels are one of the promising alternatives to this problem [2]. They have gained attention not only due to their potential of ensuring energy supply, but also due to the fact that net GHGs (greenhouse gases) emission by the use of biofuels is nearly zero [3]. Bioethanol, the ethanol produced from biomass, is one of the main types of biofuels being produced commercially. It may be produced from sugar- or starch-rich food crops (known as “*first generation biofuels*”) such as cereals, sugarcane, sugar beet, and corn or lignocelluloses and organic waste materials (known as “*second generation biofuels*” or “*cellulosic ethanol*”). Brazil and USA, world's largest ethanol producers, together accounting for more than 65% of global ethanol production, produce ethanol from sugar cane and corn, respectively [4]. But the sustainability of first generation biofuels has been criticized because, beyond a threshold point, biofuel production form food crops is not possible without threatening food supplies and biodiversity. Therefore, second generation biofuels, produced from low cost substrates [2] such as nonfood crops and nonedible parts of the food crops, make good candidate for dependence for major energy supplies in the future. The process of cellulosic ethanol production has been outlined in Figure 1.

**Figure 2:**
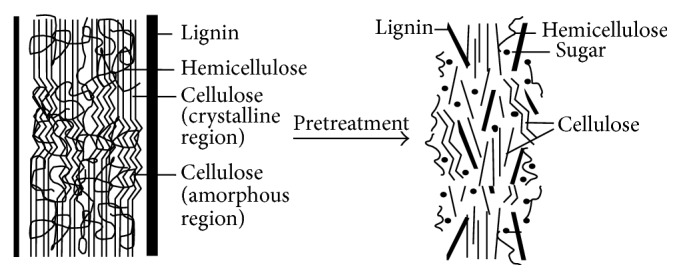
Effects of pretreatment.

Lignocelluloses account for 50% of the biomass in the biosphere [5]. The diversity of lignocellulosic feedstock [6] and its abundance also eliminate the problem of competition for feeding and fueling from food crops [7]. Lignocellulosic biomass is mainly composed of cellulose, hemicellulose, and lignin [8]. The celluloses and hemicelluloses are the polymers of fermentable sugars. They are arranged in a complex structure in close association with relatively recalcitrant, noncarbohydrate polymer of lignin and are not easily accessible for their hydrolysis. A pretreatment step is, therefore, required to alter the structure of lignocellulose. Pretreatment opens up the structure of lignocellulose (Figure 2, [9]) by partial breakdown of its constituent polymers, weakening of lignin and hemicellulose heteromatrix and reducing the crystallinity of cellulose [9]. As a result, pore size is increased and cellulosic and hemicellulosic surface areas are exposed for enzymatic or chemical saccharification to sugar monomers.

The pretreatment increases the yield of hydrolysis products to about 90% against less than 20% in the untreated biomass [10]. The pretreatment can be done by using various physical, chemical, and biological methods or combination of these methods (Table 1).

Among various physical methods, irradiation is an attractive method for pretreatment. In biomass irradiation process, biomass is exposed to high energy radiations such as ultrasonic waves, microwaves, *γ*-rays, and electron beam. The effectiveness of the treatment depends on several factors such as frequency of radiations, time of exposure, composition of the biomass, and resistance to the radiations by medium between radiations and biomass. The high energy radiations increase the specific surface area of biomass; decrease the degrees of polymerization and crystallinity of cellulose; and partially hydrolyze the hemicellulose and lignin components [11].

## 2. Gamma Irradiation

Gamma radiations (*γ*-rays) are very high energy radiations consisting of high energy photons, with deep penetration power, and are produced by the decay of atomic nuclei as they return from high to low energy state (“*gamma decay*”). They are one of the ionizing radiations of electromagnetic spectrum, capable of producing ionization cascade in matter. The radioactive nuclides of Cobalt-60 and Cesium-137 produce gamma rays spontaneously when undergoing self-disintegration [12]. For irradiation by cobalt, ^60^Co is loaded into a sealed chamber of metal alloy to prevent escape of the rays [13]. Radiations then travel from the sealed source at the speed of light and bombard the biomass. The energy carried by the gamma radiation is transferred to the biomass components by collisions of radiations with their atoms. Equal energy is absorbed by the carbon, hydrogen, and oxygen atoms in the biomass polymers. The collisions result in the loss of electrons by atoms causing their ionization. Various short- and long-lived radicals are formed. The biomass structure is altered by cross-linking and molecular scission in the lignocellulosic polymers [14]. Starch and cellulosic and pectic polysaccharides are degraded by cleavage of glycosidic bonds [13]. High doses of gamma rays depolymerize or delignify the cell wall constituents [15, 16]. Loss in the fiber content has been observed in plant matter [17]. A reduction in the rumen dry matter has been found in spruce sawdust, barks of spruce, pine, and larch when irradiated with high doses of gamma rays due to the solubilization and partial breakdown of the dry matter [18]. The degradation of cell wall has been reported to show increase in digestibility of the organic matter [16] as evidenced in rice straw [19], barley straw, pea straw, sugarcane bagasse, sunflower hulls, and pine sawdust [20]. Combined NaOH and gamma radiation treatment of wheat straw, cotton shells, peanut and soybean shells, and extracted olive oil cake and extracted unpeeled sunflower seeds showed improved digestibility compared to the individual methods [21]. Increased digestibility suggests the potential of gamma irradiation in pretreatment step during cellulosic ethanol production. Gamma radiations induced mutants with high lignolytic activity in* Pleurotus sajor cajo,* due to increased MnP enzyme production, can be utilized in biological delignification of lignocellulosic biomass [22]. In* Brachypodium distachyon* also, irradiation with 200 Gy gamma rays has shown enhanced expression of lignolytic genes resulting in degradation of lignocelluloses [23].

Gamma pretreatment enhanced the acid and enzymatic hydrolysis of biomass in bagasse [24], rice straw, rice hull, and corn husk. Increased sugar yields have been observed when wood chips, paper, grain straw, hay, and kapok irradiated at higher levels up to 1.7 to 2 MGy followed by their saccharification with cellulase enzyme [25]. Growth enhancement in cellulolytic microbes, such as* Myrothecium verrucaria* fungus, has been noticed in irradiated rice straw [26]. A marked increase has been witnessed in hydrolysis yield of gamma irradiated* Khaya senegalensis* and* Triplochiton scleroxylon* [16]. Efficiency of gamma irradiation is manifested in its capability of increasing enzymatic hydrolysis of rice straw even at high substrate loadings [27] and improved pretreatment results in comparison to steam explosion method [28].

The effectiveness of gamma pretreatment can be increased by combining irradiation with other pretreatment methods, physical as well as chemical. Combined methods enhance each other's effect, making pretreatment more efficient at relatively milder conditions. During pretreatment of rice straw, coupling of milling, autoclaving, and gamma irradiation (70 Mrad) increased the yield of total reducing sugar after pretreatment and saccharification during ethanol production [29]. Similar results were shown by wheat straw when treated using gamma rays along with crushing [30]. A study by Helaln [31] indicated increase in the contents of reducing sugar (4-fold) and soluble and crude protein after pretreatment of rice straw with gamma radiations followed by its saccharification with* A. ochraceus*,* A. terreus*, or* T*.* koningii*. The required dose of gamma irradiation was reduced from 500 kGy to 10 kGy when wet straw was used instead of dry straw. Similarly, enzymatic saccharification of sawdust, rice straw, and sugar cane bagasse demonstrated relatively larger reducing sugar release from substrates pretreated with alkali (0.1 g/g) and 500 kGy gamma rays compared to either method alone [32]. An integration of urea and gamma irradiation has shown reduction in fibre content and increased digestibility of wheat straw, cotton seed shell, peanut shell, soybean shell, and extracted olive cake and extracted unpeeled sunflower seeds [21] and enhanced reducing sugar yield in Thai rice straw and corn stalk [33]. Gamma irradiation during acid pretreatment has enhanced saccharification yield in chaff [34], filter paper [35], and wheat straw [36]. The increase in the enzymatic hydrolysis, after combined pretreatment, can be attributed to decrease in crystallinity of cellulose, depolymerisation of cellulose, loss of hemicelluloses, and removal or modification of lignin [37]. The degree of cellulose degradation varies with the nature of the biomass and environmental conditions during irradiation [26]. However, it has no direct relation with either the cellulose or the lignin content [27].

Gamma rays have also raised ethanol production from orange peels due to decrease in limonene content, a fermentation inhibitor commonly found in orange peels [37].

## 3. Electron Beam

**Figure 3:**
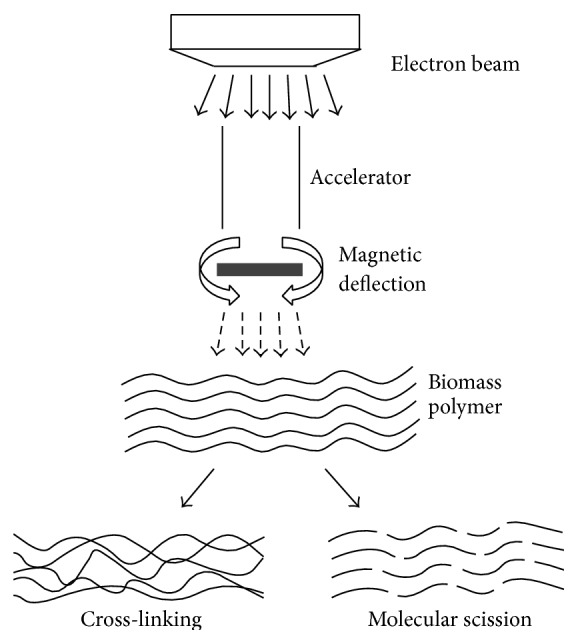
Electron beam irradiation of biomass.

Electron beam irradiation is the process of exposing target material to accelerated and highly charged stream of electrons. The kinetic energy of the moving electrons accounts for high energy carried by the beam. “*Electron beam accelerator*” is the commonly used device for electron beam irradiation. A stream of electrons is emitted from the source or an “*electron gun.*” An accelerator speeds up the electrons. Focussing is mediated and regulated by magnetic focus and deflection systems. The electron energy can be modulated by varying the irradiation dose. Bombardment of the beam with the material transfers energy carried by it directly to the material components. This eliminates the need of heating for permeation of chemicals into the material being processed [38]. The heating also initiates various chemical and thermal transformations. In biomass, electron beam irradiation shows multiple effects somewhat similar to other ionizing radiations. Studies have indicated that interaction of high energy electron beam causes depolymerisation of cellulose as a result of chain scission [39]. Polymers are also modified chemically due to oxidising effects of electron beams. Several studies have demonstrated oxidation of chemical groups and introduction of carbonyl and carboxyl groups [40, 41]. Hydrogen bonds are broken between cellulose chains making it more amorphous causing reduction in crystallinity [39]. As a consequence, mechanical strength reduction occurs, in addition to increase in the solubility and reactivity of cellulose. Irradiation also generates numerous free radicals, which further aid in structure rupturing effects of radiations. Cross-linking is also seen in the polymers upon irradiation with electron beam [42]. General mechanism of electron beam on biomass destruction has been shown in Figure 3.

Polymer chain cleavage has, however, important role in biomass pretreatment. The mechanistic effects vary with the dose of irradiation. Chain cleavage effects are particularly prominent at higher doses [42]. The destructive effects are intensified at elevated temperature [41] and in wet conditions [42].

Thus biomass modifying and rapid degradation potential of electron beams has proven an efficient method in biomass pretreatment. Immense literature support is available (Table 2), which signifies the utilization of electron beam in bioethanol production technology and other lignocellulose based applications.

Thus the positive effects of electron beams, alone or in combined methods, can be optimized for different feedstock and an advanced technology can be developed for production of bioethanol in an environment friendly manner.

## 4. Sonication

**Figure 4:**
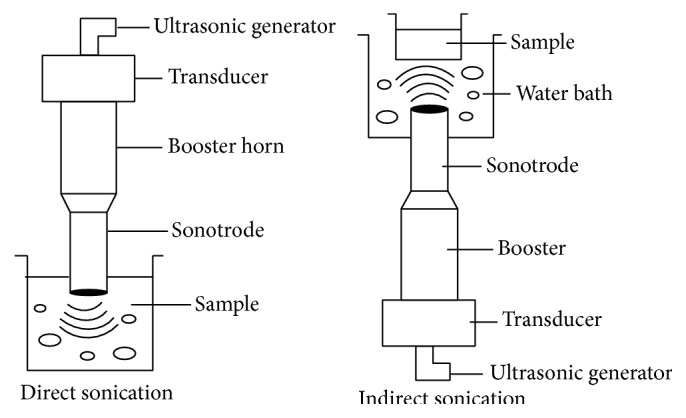
Types of sonication.

**Figure 5:**
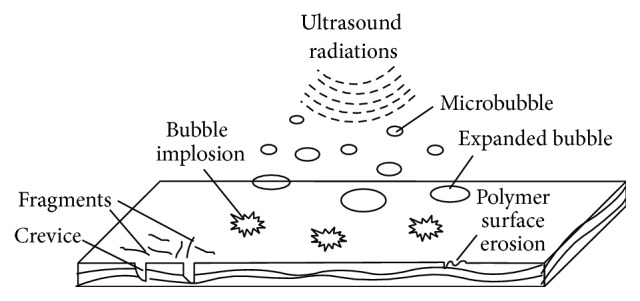
Surface modifications by sonication.

Sonication is the process of application of sound waves to a sample for a wide range of applications. When biomass is exposed to the sound waves of ultrahigh frequency, that is, 100 kHz to 1 MHz, it results in disintegration of the polymeric constituents in the biomass. Biomass is usually suspended in an aqueous medium [63] and subjected to sound-wave treatment. Two types of sonicators can be used [64]. In direct sonication, a sonication probe is directly inserted into a sample vessel, whereas indirect sonication involves a sonication bath in which sound waves are propagated through a water bath containing the sample vessel (Figure 4). The ultrasonic waves cause periodic compression and expansion of water phase. The acoustic waves are generated which show directional propagation [65]. At sufficiently high intensities, the acoustic waves cause cavitation [66] by breaking cohesion forces between water molecules. Cavitation is the process of formation and collapse of bubbles. The bubbles are filled with gases or vapours and can be stable or transient [67]. The microbubbles grow in size and become unstable and some of them implode during cyclic compression. A shock wave is propagated causing mechanical effects such as turbulence and liquid circulation. Energy is released by bubble collapse causing local increase in temperature and pressure (local hot spots) as high as 10000 K and 1000 bar, respectively [67]. These extreme conditions cause dissociation of water vapours entrapped in the cavities into OH, O, and H radicals [68]. H_2_O_2_ and HO_2_ are also formed along with a variety of other free, micro-, and macroradicals. The energy released and shear force generated are high enough to disrupt any chemical linkage at the biomass surface. A complex chemistry is rather involved in biomass degradation. Structural transformations in the biomass start at the liquid solid interface (Figure 5) and are conveyed internally through free radicals. The bonds in the aromatic rings and side groups are broken and more macroradicals are formed [64]. Radicals from sonolysis of water and biomass components together promote hydrolysis of polymers. Interactions between lignin and hemicellulosic components are affected selectively. This weakens the cell wall complex and opens its structure. The cellulosic crystallinity is decreased and depolymerisation occurs [69]. These structural disruptions increase the surface area [70], thereby making biomass accessible for other chemical and biological modifications.

The mechanical and physical processes of ultrasound can be exploited for pretreatment of different lignocellulosic biomass [71] suggesting its potential for application in biofuel production technology. Ultrasound pretreatment has boosted the total and reducing sugars yield from bagasse [72], rice straw [73], and banana flower stalk [74]. Sonication of wheat straw followed with acid hydrolysis has resulted in 132.96 mg/g of sugars due to structural disruption compared to 24.69 mg/g in control without ultrasound treatment [75]. Several factors such as exposure time, frequency during sonication, temperature during process, type of biomass, solvent used, and sonicator design decide the efficacy of the pretreatment [64, 76].

Reports have indicated degradation of a variety of poly- and disaccharides such as starch, cellulose, lactose, sucrose, and dextrans with ultrasonication [68]. Hemicellulosic and lignin components can be removed selectively using ultrasound mediated lignocellulosic fractionation depending on the sonication conditions for a specific feedstock. Dewaxed bamboo culms [77] and various other lignocellulosic substrates [63] subjected to ultrasound assisted extraction using different solvents have enhanced lignin expulsion from biomass. Both the recovered lignin and the residual solid biomass can be utilized for production of different value added products. The extracted lignin is relatively thermostable [77] and its structure is preserved during extraction [63]. Compared to conventional methods, ultrasound waves have extracted larger hemicellulosic fraction from corn cob [78] and buckwheat hulls while retaining its structure and biological activities [79].

Integration of sonication with other pretreatment methods shows significant improvement in biomass pretreatment. 9.2% higher hemicellulose, with higher purity and molecular weight, has been recovered from dewaxed wheat straw when sonicated for 35 min with 0.5% NaOH in 60% methanol [80]. Up to 50% of the relatively pure and stable lignin removal has been achieved from dewaxed wheat straw pretreated with 0.5 M KOH, at 35°C along with ultrasound exposure for 35 min [81]. Sonoassisted alkali pretreatment of sugarcane bagasse has removed 74.44% of lignin, yielding 69% hexose, 81% pentose, and 0.17 g/g of ethanol in the saccharification and fermentation steps [82]. The delignification was achieved in lesser time at relatively milder temperature conditions and generated very less amount of inhibitors [83]. Ultrasound has enhanced pretreatment effects in cotton gin trash [84], saccharification yield in garden biomass [85], and ethanol yield in sugarcane bagasse (0.38 g/g) [86] when combined with alkali pretreatment method. Coupling of acid pretreatment with sonication has resulted in higher sugar recovery from rice straw [87] and bagasse (94% of the expected yield) [88].

Combined ultrasound and ionic liquid treatment has proven an efficient method of pretreatment in rice straw using choline hydroxide [89] and in bagasse using choline acetate [90] and 3-butyl-1-methylimidazolium chloride [91]. Effect of ammonia pretreatment has also been augmented by sonication in sugarcane bagasse resulting in 95.78% cellulose recovery and 58.14% delignification, with 16.58% glucose extraction upon hydrolysis while generating lesser amount of inhibitors [92]. Sindhu et al. [93] have demonstrated significant rise in sugar concentration (0.661 g/g) from sugarcane tops when pretreated with surfactant assisted sonication.

Structural analysis has revealed processing of lignocellulosic biomass as a result of surface erosion during sonication [93]. The enhanced sugar release has been attributed to effective transport of sugar molecules due to strong convection generated during sonication [73]. Other factors contributing to sonication effects include increased mass transfer rate and water diffusion and decreased cellular adhesion [94]. Alone sonication destructs the cell wall structure, increases wood permeability coefficient, and forms microscopic channels [94]. However, integration with other methods accelerates pretreatment reactions, reduces processing time, converts biomass components selectively, and benefits in process economics.

Besides the pretreatment, cost of cellulosic ethanol production is also affected by saccharification and fermentation steps. Reports have shown marked influence of microwave irradiation on intensification of saccharification [95] and fermentation [96] output. Productivity enhancement claimed in fermentation during separate hydrolysis and fermentation is possibly due to modification in cell envelope of ethanologenic microbe without cellular disruption [96]. Implementation of ultrasound has also shown substantial stimulation in ethanol production during simultaneous saccharification and fermentation [97].

## 5. Microwave

**Figure 6:**
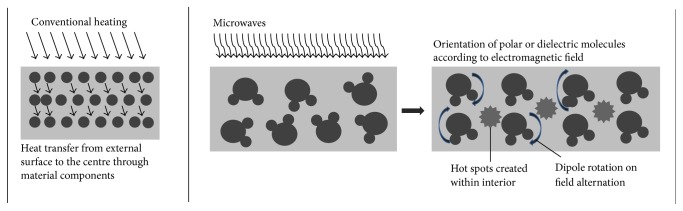
Conventional and microwave heating mechanisms.

Microwaves are the electromagnetic waves with frequency in the range of 300 MHz to 300 GHz [98]. In cellulosic ethanol production process, microwaves are employed in pretreatment and saccharification steps. Pretreatment of lignocellulosic biomass by microwave irradiation is based on nonthermal and thermal effects of microwaves. Heating is very essential parameter in pretreatment technology. Higher temperature accelerates the reaction rate and minimizes the chemicals requirement during pretreatment. Heating mechanism of microwaves makes it a preferred method over conventional heating. Conventional heating requires transfer medium, starts at the contact surface, and is conveyed inwardly by diffusion. Overheating on external side is a common problem with it. Microwave heating, however, heats entire volume simultaneously without direct contact with the material and thus renders uniform, rapid, and volumetric heating. Heating with microwave can also be regulated instantaneously [99]. Microwave heating is also called* “dielectric heating,”* which works on interaction between polar molecules or electrically conductive, dielectric chemical species and oscillating microwave electromagnetic radiations. The dielectric molecules align themselves with the electric and magnetic field of microwaves. Oscillating field causes agitation and alternation causes rotation of molecules (*dipole rotation*). Heat is generated consequently. Dielectrics present in lignocelluloses include water, cellulose, hemicellulose, and organic acids [100]. Heating creates hot spots within lignocelluloses, which shows an explosive effect on the recalcitrant structure rendering its disruption.  Figure 6 depicts the mechanism of microwave heating and its comparison with conventional heating.

Different feedstocks show varying susceptibility to microwave induced alterations subject to their chemical composition [101]. Biomass with different composition varies in its dielectric properties [102]. Microwave radiations also exhibit nonthermal effects [103] such as molecular mobility, plasma formation, and enhanced diffusion in solids, which collectively contribute to structural disruptions. Though existence of nonthermal effects of microwave is debatable, its effects seem to be less pronounced than that of the thermal effects.

Dynamic alterations are seen in microwave irradiated lignocelluloses. Thermodestruction of lignin, hemicellulose, and cellulose is observed [104]. Porosity of biomass increases as a result of volatilization, allowing higher diffusion of oxygen. Mass loss also occurs till a threshold exposure time, beyond which no more vaporization takes place [105].

Utilization of microwaves in cellulosic ethanol production has been illustrated in the vast literature of various research studies. Microwave irradiation in sawdust in* C. deodara* modified the cell wall structure and exposed hemicelluloses, which resulted in high xylanase production by* Geotrichum *sp.* F3* fungus [106]. Nonthermal effects of microwaves exhibited through plasma formation indicated erosion of lignin layer (*plasma etching effect*) in sugarcane as analysed by mass spectroscopy and Fourier-Transform Infrared Spectroscopy (FT-IR) [107]. Comparison of microwave heating with sand bath heating in corn stover has shown faster removal of xylan, lignin, and acetyl and increase in biomass digestibility. Elucidation of cellulose structural alterations displayed breakdown of amorphous regions [108]. Extensive exploitation of microwave effects has been done especially in enhancing the efficiency and reducing process time during pretreatment of biomass by other physicochemical methods. Microwave irradiation at 250 W for 10 min has dramatically increased the reducing sugar yields in switchgrass soaked in 3% NaOH solution [109]. The application of combined microwave and H_2_O_2_ activated ammonium molybdate pretreatment of woody biomass yielded 59.5% of sugar [110]. Two-stage pretreatment of sugarcane bagasse using 1% NaOH followed by 1% H_2_SO_4_ in microwave irradiated environment has been reported to enhance fermentable sugar extraction to 0.83 g/g of dry biomass. 90% lignin removal has been achieved by microwave-NaOH treatment at 450 W for 5 min [111]. Chen et al. [112] have demonstrated 80–98% of hemicellulose extraction from bagasse pretreated with combined acid-microwave pretreatment. Microwave assisted dilute ammonia pretreatment in sorghum bagasse removed 46% lignin, which resulted in increased porosity of biomass. This in turn enhanced glucose and ethanol yields to 42/100 g and 21/100 g dry biomass, respectively [113]. Lignin and hemicellulose extraction from KOH-microwave pretreated corn cob has also shown increase in surface area resulting in production of 34.79% sugars in hydrolysis [114]. Significantly high reduction in hemicellulosic, cellulosic, and lignin contents was observed in trunk and fronds of* Elaeis guineensis* when pretreated with microwave-alkali methods [115]. Microwave assisted alkali and acid pretreatment of oil palm empty fruit bunch has shown 71.9% reduction in lignin content [116]. Substantial amount of glucose concentration (48.58 g/L) was produced from corncobs pretreated with microwave combined with alkali-acid method [117]. Integration of microwave with H_2_O_2_ pretreatment in rice straw exhibited cell wall rupturing by disruption of silicon waxy structure and breakage of ether linkages between lignin and carbohydrates resulting in removal of lignin and increase in crystallinity index to 63.64% and 1453.64 *μ*g/mL of sugar production upon enzymatic saccharification [118]. Integration of acid [118] and alkali [119] with microwave pretreatment has also shown similar results. Sugarcane tops have been successfully saccharified yielding 376 g/g sugars after surfactant aided microwave pretreatment [120]. Water hyacinth subjected to microwave-dilute acid pretreatment augmented saccharification yield up to 94.6% of expected theoretical value, as a result of hemicellulose breakdown [121]. In corn straw and rice husk, marked enhancement was observed in hydrolysis with enzyme from* Myceliophthora heterothallica*, when pretreated with combined microwave and alkali-glycerol method compared to unirradiated biomass [122]. Zheng et al. [123] have found enhanced sugar recovery from microwave-glycerol pretreated corncob as a consequence of selective removal of lignin and hemicellulose fractions during pretreatment. Thus conventional heating can be replaced with microwave heating during biomass pretreatment owing to speed acceleration capability of microwaves at the same temperature. This may contribute to improved economic feasibility of the process.

A study by Nomanbhay et al. [124] has indicated 5.8-fold increase in microwave assisted enzymatic hydrolysis in oil palm empty fruit bunch fibre suggesting positive role of microwave in saccharification. Enhanced enzymatic saccharification has also been observed in other research studies [125, 126].

## 6. Advantages of Irradiation

Characteristics of radiations can be exploited in all steps of bioethanol production, that is, pretreatment, saccharification, and fermentation, directly or indirectly. The aim of the pretreatment is to disrupt the tough lignocellulose complex to expose utilizable polymers. Conventional methods of pretreatment count on chemical or physicochemical methods primarily. But certain limitations associated with them need to be overcome, which has diverged the technological innovations to other advanced techniques. Irradiation is equally effective in biomass degradation as other methods, with additional advantages of controlled selective degradation of biomass components unlike chemical methods which sometimes lead to loss of some cellulosic or hemicellulosic parts. Irradiation does not involve use of solvents in large quantities eliminating need of their recovery or recycling. The problem of corrosion associated with some chemicals is not faced during irradiation. One big challenge of reduction of fermentation inhibitors generation is addressed well in radiation treatment [53]. The energy input in terms of heat required for chemical penetration is avoided [38]. Downstream steps of cooling and neutralization after biomass pretreatment are also not needed [49]. The comparison with biological pretreatment, however, is in progress as research is ongoing for both irradiation and biological methods. In addition, the saccharification enhancement effects of sonication [127] and tolerance enhancement induced by mutation potential of radiations in ethanologenic microbes [128] further broaden the scope of irradiation in the process of bioethanol production.

## 7. Economic Feasibility

Capital cost and operational cost are important parameters in determining the efficacy of a pretreatment method. The research studies involving irradiation of biomass need to be elaborated to assess the economic viability of the method for various feedstocks. Laboratory scale studies have just validated the potency of radiations in biomass pretreatment and indicated high capital cost because of special bioreactors required in the process. Also the operational cost involving high energy radiations is quite high. So adoption of irradiation as sole pretreatment method especially at industrial scale seems economically infeasible. However, the advantages associated with radiations cannot be ignored. Therefore, different approaches can be used to minimize the cost of pretreatment. The capital cost can be minimized, though over a short range, by designing high efficiency bioreactor with the help of engineering expertise. The operational cost can be reduced significantly across relatively wider range by combining irradiation with inexpensive chemical pretreatment methods or subjecting biomass to two-stage pretreatment which will be less energy intensive than individual method. Lower dose requirements, shorter duration involvement, and moderate process conditions can prove beneficial in irradiation combined with other physicochemical methods. Alternatively, the cost of upstream and downstream processes can be reduced so that the overall cost of bioethanol production process may not vary significantly. This can be achieved successfully by maximizing saccharification yield using highly catalytic hydrolases, utilizing advanced techniques of simultaneous saccharification and fermentation or simultaneous saccharification and cofermentation, and also generating various value added products by biorefinery.

## 8. Important Considerations

Owing to the advantages of irradiation, advanced technologies can be established for biomass pretreatment and downstream processing. However, certain points are to be considered beforehand. The research studies conducted till now have focused on biomass irradiation at lab scale only. Pilot scale studies are required to validate the outcome. The commercial implementation is a costly affair which needs to be taken care of during technology development. Furthermore, certain safety regulations are to be followed while using radiations to avoid health hazards associated with them.

## 9. Future Prospects

Irradiation is a recent and relatively less explored approach. Research studies are limited and can be elaborated. Optimization studies are essentially required for specific feedstock for broadening the prospects of irradiation applicability. Scale-up studies propose expansion in research scopes of role of irradiation in production of biofuel. At the end, commercialization is envisioned in the future as integrated part of biofuel production technology.

## 10. Conclusion

The indispensable beneficial effects of irradiation can be used in bioethanol production from lignocelluloses in improving the results of both pretreatment and saccharification steps. The effectiveness of radiations pretreatment is advantageous as it brings reproducible and quantitative changes in the biomass characteristics. The reactions can be commenced at moderate conditions of temperature and lesser amounts of chemicals are needed in methods integrated with other chemical treatments. The reduced energy input demands and other factors collectively can make overall process economically feasible. Radiations are thus a unique source of energy and their technological implementations can provide simpler, efficient, cost effective, and ecofriendly methods in biofuel industry.

## Figures and Tables

**Table 1:** Methods of pretreatment of lignocellulosic biomass.

Pretreatment	Advantages	Disadvantages
**Physical**		
Milling, chipping, shredding, grinding, irradiation, and pyrolysis	Increase in biomass surface area & pore size, no requirement of chemicals, and depolymerization & reduced cellulose crystallinity	Highly energy intensive & industrially inapplicable as individual methods

**Chemical**		
Dilute acid pretreatment Concentrated acid pretreatment	Lesser acid is needed Efficient, low temperature required	Corrosive & formation of fermentation inhibitors (furfurals)
Alkali pretreatment	Lesser inhibitors formation	Less effective for lignin-rich biomass

**Physicochemical**		
Steam explosion AFEX (Ammonia Fiber Explosion) Method SO_2_ and CO_2_ explosion	Lesser retention time No inhibitors formation, can process coarse biomass More effective than AFEX	Xylan & lignin degradation Less effective for lignin-rich biomass Unaltered lignin & hemicellulose
Liquid Hot Water (Aquasolv)	No catalyst required	High water requirement
Wet oxidation	Rapid	Fermentation inhibitors
Ozonolysis	No inhibitors formation	Very expensive
Organosolv	Pure lignin extraction	Costly & solvent inhibition
Oxidative delignification	Rapid, low temperature needed	Solvent recycling needed
Ionic liquids	High biomass loading processing	Solvent recovery required

**Biological**		
Using lignolytic (white, soft and brown rot) fungi and actinomycetes	No inhibitors generation, no chemical or harsh conditions required, and low energy requirements	Very slow rate, at experimental stage

**Table 2:** Effects of electron beam on lignocellulosic biomass.

Biomass	Treatment conditions	Structural alterations	Results	References
Waste papers	Electron beam treatment integrated with gamma irradiation	⋯	Increased rate of hydrolysis	[43]

Wood chips	30 kGy electron beam pretreatment	⋯	Reduced strength, reduction in energy consumption to 20–25% for high yielding pulp	[44]

Rice straw	NaOH assisted electron beam pretreatment	Reduction in size of rice straw	Increased sugar content compared to that in individual methods	[45]

Rice straw	Electron beam irradiation	Surface changes in biomass	Increased digestibility, 65.5% of theoretical sugar yield, and no inhibitory products formation	[46]

Rice Straw	Alkali-electron beam irradiation	Reduction in lignin content, increase in cellulose content from 19.5% to 64%	Enhanced sugar yield in hydrolysis	[47]

Rice straw	Beam irradiation combined with 3% dilute acid treatment followed with autoclaving	⋯	80% total sugar yield	[48]

Rice straw	80 kGy beam irradiation	⋯	52.7% ethanol yield after simultaneous saccharification and fermentation with *Mucor indicus*	[49]

Wheat and rice straw	Electron beam treatment at 200 Gy	⋯	More delignification by *Phanerochaete chrysosporium, *more cellulase production	[50]

Wheat straw	Electron beam treatment comparison: single irradiation at 100 kGy and divided irradiation at 25 kGy in 4 tandem doses	Reduction in cellulose crystallinity from 43% to 38.8%, removal of hemicellulose, and lignin modification	74.9% saccharification yield upon single irradiation compared to 40.9% in control and 51.1% in divided irradiation	[51]

Wheat straw	Electron beam pretreatment	Reduction in dry matter	Increased degradability	[52]

Sugarcane bagasse	Electron beam irradiation at 50 kGy followed with dilute acid and hydrothermal treatment	⋯	30% enhancement in enzymatic saccharification by irradiation in hydrothermal treatment compared to 20% in acid treatment	[53]

Sugarcane bagasse	Electron beam irradiation	Breakage of bonds in lignocellulosic matrix	Dose-dependent increase in fibre degradation and increased rumen digestibility	[54]

Kenaf core	500–1000 kGy beam irradiation and autoclaving for 5 hrs	⋯	Increase in crystallinity index from 50.65 to 555 at 500 kGy, gradual increase in sugar concentration from 100 to 500 kGy being 83.9% at 500 kGy	[55]

Kenaf core (*Hibiscus cannabinus*)	Combined alkaline-electron beam method	⋯	72.4% total sugar recovery in hydrolysis with 63.9% glucose	[56]

Spruce wood	2 Mgy beam irradiation	90% cellulose recovery	80% recovery of glucose with *Trichoderma* cellulase	[57]

Sawdust & chaff	100 Mrad beam irradiation	⋯	Linear increase in enzymatic hydrolysis rate with irradiation dose, reduced time of pretreatment	[58]

Hybrid poplar	Beam irradiation followed with mild alkali extraction	Xylan degradation	Enhancement in extraction and enzymatic hydrolysis with commercial cellulase	[59]

Oil palm empty fruit bunch	Electron beam irradiation at 400 kGy	Biomass degradation; cellulose, hemicellulose, and linin deformation	Reduced crystallinity index, increased solubility in water, benzene, and NaOH	[38]

Bamboo	Beam irradiation from 0 to 50 kGy	Cellulose structure alteration	Gradual decrease in crystallinity index with increasing dose	[42]

Switchgrass (*Panicum virgatum* L.)	1000 kGy beam irradiation followed with hot water extraction	Decreased cellulose crystallinity, decrease in hemicellulose content from 32.2% to 16.9%	Decrease in molecular weight, 4-fold increase in glucose yield	[60]

Pistachio byproduct	Beam irradiation at 30–40 kGY	Reduction in ADF, NDF and increase in ADL	Decreased tannin content, enhanced fermentation	[61]

*Miscanthus sinensis*	Beam irradiation at 500 kGy Beam irradiation with aqueous ammonia treatment	⋯	1.26-fold increase in saccharification compared to control, 2.4-fold increase in combined treatment, and production of 96.8% ethanol from hydrolysate	[62]
